# Utilization of dietary glucose in the metabolic syndrome

**DOI:** 10.1186/1743-7075-8-74

**Published:** 2011-10-26

**Authors:** Marià Alemany

**Affiliations:** 1 Department of Nutrition and Food Science, Faculty of Biology, University of Barcelona, Barcelona, Spain; 2 CIBER Obesity and Nutrition, Institute of Health Carlos III, Spain

**Keywords:** metabolic syndrome, insulin resistance, adipose tissue, hyperlipidemia, glycolysis, glucose fate

## Abstract

This review is focused on the fate of dietary glucose under conditions of chronically high energy (largely fat) intake, evolving into the metabolic syndrome. We are adapted to carbohydrate-rich diets similar to those of our ancestors. Glucose is the main energy staple, but fats are our main energy reserves. Starvation drastically reduces glucose availability, forcing the body to shift to fatty acids as main energy substrate, sparing glucose and amino acids. We are not prepared for excess dietary energy, our main defenses being decreased food intake and increased energy expenditure, largely enhanced metabolic activity and thermogenesis. High lipid availability is a powerful factor decreasing glucose and amino acid oxidation. Present-day diets are often hyperenergetic, high on lipids, with abundant protein and limited amounts of starchy carbohydrates. Dietary lipids favor their metabolic processing, saving glucose, which additionally spares amino acids. The glucose excess elicits hyperinsulinemia, which may derive, in the end, into insulin resistance. The available systems of energy disposal could not cope with the excess of substrates, since they are geared for saving not for spendthrift, which results in an unbearable overload of the storage mechanisms. Adipose tissue is the last energy sink, it has to store the energy that cannot be used otherwise. However, adipose tissue growth also has limits, and the excess of energy induces inflammation, helped by the ineffective intervention of the immune system. However, even under this acute situation, the excess of glucose remains, favoring its final conversion to fat. The sum of inflammatory signals and deranged substrate handling induce most of the metabolic syndrome traits: insulin resistance, obesity, diabetes, liver steatosis, hyperlipidemia and their compounded combined effects. Thus, a maintained excess of energy in the diet may result in difficulties in the disposal of glucose, eliciting inflammation and the development of the metabolic syndrome

## Review

### Diet and the availability of nutrients

Present-day humans are adapted to eat a varied omnivorous diet, in which starches provide the largest share of energy; body physiological systems are adapted to this diet, coincident with that consumed by primitive humans [1,2]. Through evolution, we have evolved mechanisms to maximize the use of available food energy, largely plant material, and to store part of the energy as lipid for use in periods of scarcity. However, most of today's diets do not conform to this pattern, largely because of the application of the same mechanisms of food selection that helped us survive [3,4]: The current availability of high-energy lipid-rich diets, containing additional high-biological quality protein, is compounded by cortical signals inducing their consumption because of their palatability [5] and atavistic traits of craving for food containing scarce and/or high-energy nutrients [6,7]. Consequently, energy intake tends to be higher than that needed to fulfill the energy -and plastic- nutrient needs, creating a floating excess of nutrients, which should be processed and disposed of. The physiologically established mechanism for excess nutrient energy removal is thermogenesis [8-10], carried out by the uncoupling protein system of brown adipose tissue (BAT) [11], but also by thyroid-induced organ insufficiency [12,13], increased energy expenditure through protein turnover, and other metabolic activities [14,15]. These mechanisms tend to reduce the excessive buildup of dietary fatty acids and glucose, but are of only limited effect on excess amino acids because of the additional need to remove amino nitrogen.

The elimination of excess glucose also poses a few problems by itself. Glucose is the main inter-organ energy staple, and is released in large amounts by the intestine-portal vein-liver system because the main dietary component is supposed to be starch, which digestion yields glucose. The absence of dietary glucose is a physiological signal in itself, a correlate of starvation, and elicits the mobilization of lipid stores [16,17] to cover the body energy needs. Thus, high lipid availability is construed as a sham "starvation-like condition", which prevents the massive oxidation of glucose [18]. Evidently, excess lipid availability because consumption of high-fat diets is not akin to real starvation or energy deficit, but the preservation of glucose stands; furthermore, high glucose and energy availability enhance the protection of dietary amino acids (paradoxically also in excess) from their utilization as energy substrates [19,20].

The combination of excess fatty acids and excess glucose poses a serious problem to the homoeostatic maintenance of energy balance, a condition unique to the metabolic syndrome (MS) [21,22]. The body has to find ways to circumvent the strict glucose preservation measures painstakingly developed and established through evolution for its own protection, such as insulin resistance [23,24].

In the present review, these processes and effects are shown as both homoeostatic control systems and pathogenic mechanisms in the development of the metabolic syndrome,

### Excess glucose and insulin resistance

After insulin resistance denies its entry to muscle, and decreased blood flow restricts adipose tissue uptake, most of the remaining glucose could only be used in significant amounts by BAT (to sustain thermogenesis and for lipid storage) [25], or by the liver, the intestine and -perhaps- by the microbiota. The liver capacity to eliminate excess glucose is limited because of space availability constrictions to glycogen and lipid storage [26]. However, lipogenesis has to be carried out, even countercurrent, because of an already large excess of dietary fatty acids and triacylglycerols, temporarily stored in the liver, waiting for their eventual release as VLDL. A large excess of non-exported energy (lipid, glucose) may help induce liver steatosis, damaging liver function [27]. High insulin helps drive excess glucose towards lipogenesis [28], but the process is also limited by the already excessive availability of acetyl-CoA, which cannot be converted into ketone bodies via 3-hydroxy-3-methyl-glutaryl-CoA because the high levels of glucose fully inhibit the ketone pathway [29]. As a consequence, glucose levels keep rising and/or are maintained high. Let's look now where the unwanted glucose may go.

BAT enhanced consumption of glucose may represent a quantitatively significant possibility for rodents, but it is doubtful that in humans, with a limited BAT presence [30,31], it may represent a significant dent in the pool of excess circulating glucose, especially when BAT preferred substrate is, again, lipid [32].

Excess glucose becomes a danger by itself: can affect water balance because of its osmotic properties [33], and increase the glycation (and consequent loss in function) of a number of proteins, especially those in contact with the bloodstream [34]. Thus, over a certain limit, excess glucose may be lost via urine. However, before these drastic measures are taken, the body tries to correct glycemia using the whole set of instruments devised to maintain glycemic homoeostasis. High glucose levels decrease appetite [35] and thus limit the intake of food (in the end, of glucose). However, this effect is largely dependent on insulin levels and function [36], which is in turn affected by excess lipid and other insulin resistance-inducing factors [37].

Glucose entry into most cells is controlled by insulin, thus alteration of its function starves cells from access to glucose, even in front of high blood glucose, and altering tissue glycogen [38]. Control of glucose utilization by the liver relies not in transport, but on its phosphorylation, in a way that regulation of glucokinase is critical [39], but the insulin-controlled catabolism of hexoses through the glycolytic and pentose-phosphate pathways to finally -and irreversibly- yield acetyl-CoA are also critical [40,41]. The ability of the liver to store glucose as glycogen or to transform it into acetyl-CoA (for oxidation of lipid synthesis) is limited, and cannot cope with the excess glucose left over by the preferential consumption of lipid. Where goes, then the postulated excess of glucose?

Diabetes (type 2) and its principal cause/symptom, insulin resistance, are widely considered the core pathological trait defining the metabolic syndrome [42]. This is because insulin resistance increases both glycemia and insulinaemia, favoring the deposition of fat [43], and increasing the circulating lipids [44] which help raise arterial tension when combined [45]. These diseases are complementary and act synergistically in deranging the metabolic control of energy utilization [46].

Insulin resistance is closely related to excess fatty acid availability [47,48], and facilitates the deposition of fat in adipose and other tissues [49]. Muscle insulin resistance is in fact a defensive mechanism converted in a deadly trap by excessive availability of energy. Under normal conditions of limited energy availability, glycemia is low (scarce supply) and in consequence, glucose is not taken up by most tissues, being reserved for nerve tissue [50] and glycolytic red blood cells [51]. Under these conditions, lipids from the body reserves are mobilized, and ketone bodies [52] and fatty acids are made available (NEFA or fatty acids released by lipoprotein lipase activity) to the muscle. Their presence inhibits the insulin signaling cascade [53] which limits the release of GLUT4 rafts to the cell surface [54], thus effectively diminishing glucose uptake in favor of fatty acids [55].

Under conditions of excess energy available (i.e. abundant fatty acids and glucose), the effects exerted by fatty acids are the same. Consequently, the unused glucose (there is no need now to preserve it) builds up in blood. Hyperglycemia elicits the secretion of insulin by the pancreas; the ensuing hiperinsulinemia tries to counter the muscle insulin resistance. However, the combination of hiperinsulinemia and insulin resistance facilitates/induces the entry of glucose in tissues less protected than muscle. High insulinemia and high glycemia persist because most of the blood glucose, remains unused, has no place taking it up and using it in quantity. In rodents, this excess glucose is largely converted into fat by the liver and adipose tissue [56], thus aggravating the problem of substrate utilization by muscle and other peripheral tissues. In humans there is little lipogenesis from glucose under normal conditions [57,58], thus the problem of disposal of excess glucose is even greater than in rodents. However, the active production of 3C fragments by peripheral tissues, such as WAT [59,60], provides the liver with substrates for either gluconeogenesis in part blocked by excess glucose [61] or lipogenesis [62]. High 3C fragment availability potentiates hepatic lipogenesis [63,64] and may explain, at least in part, the increased production of fatty acids from excess glucose.

Dietary limitation of low glycemic index carbohydrates, but essentially decreased total energy input, may help improve the condition of the MS [65,66] by diminishing the excess of energy/substrates to dispose of, but also by flattening the curve of absorption of glucose from the gut, and thus decreasing the insulin response [67].

### The fate of excess glucose

Increased BAT [68] and muscle [69] thermogenesis can help eliminate a sizeable part of the excess unused dietary glucose. This may be, probably helped by the combination of limited muscle and adipose tissue glucose oxidation [70], liver utilization for lipid synthesis [28] and energy utilization, including thermogenesis [71], futile cycling and thyroid hormone-elicited loss of hepatic mitochondrial efficiency [72]. These energy-spending processes may also help diminish the amino acid load, but probably to a lesser extent because of the constrictions posed by the need to eliminate their amino nitrogen [73].

In addition to the processes presented above, other possible pathways for glucose disposal should be explored. Glucose freely diffuses across the intestinal wall (i.e. both ways) [74] and the total daily volume of digestive secretions is considerable; several fold higher than total volemia. Food-derived sugars are actively metabolized by the microbiota [75]. We can thus expect that an undetermined part of the excess body glucose may find its way into the intestinal lumen, where it may be taken up and metabolized by the microorganisms. Since the microbiota behaves as a symbiotic adjunct to our digestive system, its eventual participation in the handling of excess substrates may constitute a substantial part of their function under the anomalous conditions of excess energy availability. The "obese microbiota" has a different composition and different metabolic function than that of lean people eating the same diet [76]. There are, thus, factors -not immediately diet-related- that mark the wide differences between microbiota bacterial types of MS and normal individuals [77,77]. The NO·/nitrate/nitrite question may be a principal factor, but the probable availability of other diffused substrates and their acting as "overflow" energy sink is a question that may help explain part of our newly found adaptation to excess energy.

Thus, human bodies, non adapted to the new evolutionary challenge: excess of nutrients, have found ways to cope, albeit partially, with the problems posed by our deeply ingrained mechanisms of preservation and survival against scarcity rather than by excess itself.

Short-term adaptations: lipogenesis, lipid oxidation, fat deposition, cannot be maintained indefinitely; others (thermogenesis, turnover, growth) have also a limited span of application. Only long-term adaptations, affecting tissue structure and function, but also shifts in metabolic pathways (e.g. N_2 _excretion [78,79]) can be sustainable over a long period. However, in all cases, the adoption of these measures represents a forced operation (adaptation) of mechanisms not devised for these purposes, which bears consequences in the medium and long term by producing the changes we recognize largely as inflammation, the molecular basis of the MS.

Other compensatory mechanisms include the control of insulin secretion by amino acids [80,81], and a shift in the main insulin deactivation site. Under standard conditions, the liver breaks up a large part of the insulin it receives through the portal vein [82,83], the normal pancreatic blood outlet; however, in hyperglycemic obesity, the liver cannot remove a large portion of this insulin, with the consequence of permanently raised systemic insulinemia [84]. A number of tissues, such as white adipose tissue (WAT), however, develop the ability to deactivate a significant proportion of the insulin carried by the blood [84,85], a mechanism that protects the tissues themselves of being force-fed an unwanted and not metabolizable (because of saturation of normal pathways) load of glucose. This may help protect the adipose tissue, but overall aggravates the problem of glucose disposal and the growing contest between increasing glucose and insulin levels.

### Hepatic steatosis and hyperlipidemia

In the MS, cholesterol synthesis prevails over its uptake from the bloodstream [86]. Hepatic steatosis reduces the functionality of the liver, increasing the synthesis of cholesterol [87], whilst liver cholesterol uptake is impaired because of defective insulin signaling [88]. There is a decrease in circulating HDL-cholesterol [89] but an increase in that carried by LDL [90,91]. Liver altered function, i.e. decreased insulin removal [92] and blocked ketone body synthesis [93] indirectly favor cholesterol synthesis [94] and decreased liver uptake from plasma lipoproteins [95], which adds to the problem of hypercholesterolemia.

In liver steatosis, protein synthesis is also altered, not for lack of amino acids or energy, but because of lipid clogging and cell damage [96,97], which in turn elicits the proliferation of defense immune cells that additionally intervene in the already stretched hepatic function [98]. A probable key element in the development of liver steatosis is endoplasmic reticulum stress [99,100], since in liver, this cell organelle system exerts a number of functions [101,102], largely related with lipogenesis and the synthesis of complex lipids [103], but also the synthesis (folding) of proteins that will be later assembled with lipid in the dyctiosomes for export as circulating proteins or lipoproteins [104]. Alteration of the redox state or an unbalanced availability of nutrients, such as those constantly affecting the liver in the MS, may elicit an altered endoplasmic reticulum response, the breakup of the assembly line for lipoproteins and the accumulation of fat in the liver [105].

Excess peripheral production of free radicals and the oxidation of lipoproteins [106] may combine with the damage to the liver because of excess lipid accumulation to decrease its capacity to process xenobiotics [107]. The fairly constant presence of increased uric acid in plasma in the MS [108,109] indicates that xanthine oxidase activity is increased [110] helping sustain oxidative damage. It has been suggested that a relative deficit in minerals may help aggravate the situation, that is the case for magnesium [111] and, especially, zinc [112]. This in turn affects -in different ways- the metabolism of iron [113-115].

### Adipose tissue and hyperglycemia

In WAT there is a large production of lactate under conditions of insulin resistance elicited by excess fatty acids [116], which is another consequence of hyperlipidemia, excess lipid consumption and the presence in the system of more energy than needed (and which the human machinery is able to eliminate) [117]. Insulin resistance effectively decreases the muscle ability to take up glucose [55]; it does not affect brain glucose uptake, and neither does overload the liver, which lets glucose pass through undisturbed or uses it for lipogenesis (to add insult to injury, but to somehow limit the dangers of excess glucose) [118]. One of the few remaining sites large enough to use this excess glucose is adipose tissue, which despite being far from uniform in cell size, translating ability and metabolic activity [119] contains small but dynamic glycogen stores [120], fairly sensitive to catecholamines [121]. WAT is able to incorporate glucose from the blood even under conditions of insulin resistance [122,123]. This glucose may be used to produce more lactate (as observed in the obese) [60] to obtain the ATP needed for cell function under conditions of varying degrees of hypoxia [124]. However, a large portion of excess glucose finds its way into lipogenesis [125].

Hyperglycemia (and hyperinsulinemia) force WAT to take up a large part of the glucose waived off by other tissues. The logical path is the glycolytic conversion to pyruvate, which may be increased under hypoxic or anoxic conditions (i.e. with scarcely operative mitochondrial oxidative systems) as the main source of ATP. A small part of this glucose is stored as glycogen, which can be easily mobilized to produce glycolytic energy even under hypoxia [126]. But under these conditions, WAT generates an excess of acetyl-CoA, in part because of the operation of pyruvate dehydrogenase [127], but also from sporadic lipolysis elicited by catecholamine stimulation, such as that of exercise [128]; this process also activates the phosphorolysis of glycogen [129], increasing the availability of cytosolic hexoses-phosphate, and then of pyruvate. Acetyl-CoA could not be oxidized at a fast rate because of hypoxia and Krebs cycle (NADH) saturation. Under these circumstances: pyruvate dehydrogenase could not operate, causing a buildup of pyruvate/lactate and the release of the latter to the bloodstream [130,131], which generates an acidotic microenvironment in WAT, especially after adrenergic stimulation (even when mild, such as that induced by exercise) [132]. This lactate (and the corresponding lowering of blood pH) adds to the erythrocyte glycolysis, forcing the dissociation of oxygen from oxyhemoglobin [133].

The alteration of adipocyte (or macrophage) redox equilibrium may trigger also a situation of endoplasmic reticulum stress [134], which may compound the severe condition of the cells by further limiting lipogenesis and protein synthesis [135], and/or altering the immune response of the tissue (in the case of macrophages) [136].

Body energy expenditure is largely dependent on the flow of blood across the cells, enhancing their ability to interchange substrates, gases and other compounds with the bloodstream [137]. Thus, we must take consider the critical importance of blood flow across the ultimate energy sink that is adipose tissue. The lack of protection of WAT against the avalanche of lipids results into a massive enlargement of WAT lipid vacuoles. The hypertrophied tissue exerts pressure on itself and its surrounding structures, limiting -again- blood flow and, perhaps, further generating hypoxia [138].

However, this acute handling of a punctual excess of energy cannot be indefinitely maintained, day after day for a lifetime, since there are physical constraints to the unchecked size increase of WAT. In fact this limit is achieved at fairly different WAT sizes for different WAT sites and individuals, which means that the tale is not complete and that there exist additional factors which protect WAT from unstoppable growth to death [139]

### White adipose tissue, the last energy sink

WAT shares with BAT the ability to store large amounts of fat; their main difference being the higher oxidative capacity of BAT, and its idiosyncratic capability of wasting energy under tight nervous/endocrine control [140-142]. WAT is (as BAT) also a disperse organ [143,144], but most of WAT is concentrated in a small number of large masses, which purported main role is that of storing most of the body energy reserves in the form of triacylglycerols [145,146]. However, WAT also plays other functions, such as insulating, filling spaces, and physically protecting structures [59] controls some functions of their adjacent organs [147,148], as is the case of intermuscular, epicardial or perivascular WAT [149,150]. WAT is a rallying center for immune system cells [151,152], and provides stem cells for repair, regeneration or adaptive modulation [153,154]. However, probably its main role functions are its ability to store large amounts of fat and its direct implication in the control of energy partitioning and handling energy (together with the liver) under conditions of scarcity [146]. Location, in this case, equals specialization, since mesenteric WAT has a very direct implication in the handling of lipids absorbed by the intestine [155], whilst other depots (retroperitoneal, perigonadal, gluteal), with larger cells and lower overall metabolic activity play a role more adjusted to that of storage of lipid reserves [156]. Adipose tissue distribution shows marked sex-related differences [157,158], which hint to a role of sex steroid hormones in the modulation of the long-term manifestation of the MS.

There are two other important distinguishing points for WAT in comparison with all other tissues: first, WAT is not as protected against insulin-mediated glucose incorporation as are muscle [159] and the liver [160], since WAT is the *last stop *for circulating energy substrates (glucose, triacylglycerols). WAT has to take in what all other organs or tissues could not use, since it acts largely as energy buffer, to accumulate excess energy in times of affluence and to release them in scarcity. The second important difference is its ability to considerably increase its mass within the physiological range conditions [161]; only very large -and permanent- increases in overall WAT mass (i.e. in the lipid it contains) becomes a pathological condition: obesity.

WAT has considerable flexibility in distribution, cell types and numbers, mass, lipid content, and ability to store energy [162,163]. Its main reserves are triacylglycerols [164]. Nevertheless, -at least in the obese, with large WAT mass- it also deeply influences glucose metabolism, storing glycogen and releasing lactate/pyruvate in accordance with daily prandial cycles [59,120]. WAT is a well known source of hormones, it synthesizes and recycles estrone [165], and is able to interchange both estrone/estradiol and androstenedione/testosterone [166,167], as well as cortisol/cortisone (corticosterone/dehydrocorticosterone in rodents) [168]. WAT also produces a number of adipocytokines that control the response to energy challenges of the tissue itself and surrounding tissues [138,148], i.e. has both paracrine and endocrine secretions. WAT is the main organ synthesizing leptin [169], a small peptide hormone which controls gonadotropin secretion [170,171], inducing the preparation for full functional reproductive capacity. Leptin also plays an important role in the control of food intake [172,173] and body energy handling [174-176]. The complexity of WAT in the paracrine regulation of energy partition and its own size and cell distribution is exemplified by the presence of a complete rennin-angiotensin system [177], and the synthesis of other adipokines such as adiponectin [178,179]. Adiponectin is a powerful anti-inflammatory cytokine [179,180], which also participates in the maintenance of energy balance and substrate utilization control [181], and which effects are projected to other tissues [182,183].

WAT is one of the body tissues with highest ability to regenerate from stem cells to preadipocytes and fully developed adipocytes [184], depending on the demands for energy storage; cell size may change several-fold because of the often enormous accumulation of triacylglycerols [161], which routinely accounts for up to 85% of the fresh tissue weight [185]. WAT cell numbers can also decrease rapidly when storage space is not needed: selective apoptosis mechanisms cull down the number of adipose tissue cells [186]. This high versatility, and WAT endocrine function, help to control the mass of lipid energy stored [187-189], in order to make it available to the whole body under conditions of scarcity [161], often in a cyclic way as is the case of migratory birds [190] or the pregnancy/pre-lactation accumulation of fat in mammals [191].

WAT is also the main site of the inflammatory processes [138,192,193] that are at the root of the MS. This is due largely to the reasons indicated above: its role as key energy control player [194], but also to the fact that WAT is the last in the line to dispose of excess energy. Under conditions of plenty, WAT cannot dispose of the excess energy it is forced to store, grows in size, initiates the immune response [195], which is later amplified by invading macrophages [196,197], and thus obesity develops.

### Conclusion

Excess energy intake is primarily countered by the normal homeostatic mechanisms regulating body weight: signals eliciting a decrease in food intake combined with increased energy expenditure, i.e. higher thermogenesis, increased metabolic activity (including enhanced protein turnover), decreased overall metabolic efficiency (which may be also considered part of the thermogenic process), and, ultimately, increased energy storage. However, continued exposure to high-energy diets may either overcome the possibilities of these systems or/and erode their efficiency, resulting in unbearable excess energy accumulation on the storage depots. The capacity of body organs to store glycogen and fat are limited, and excessive buildup of reserves provokes tissue damage, forcing the intervention of the immune, albeit with little success and considerable (and largely damaging) release of metabolic control signals which compound the problem. The arrest of WAT fat accumulation is obtained at the price of loss of energy partition functionality and lefts the energy partition control system in disarray, conditions that eventually develop in a constellation of metabolic alterations that constitute the MS.

High fatty acid availability, often the consequence of high-energy diets rich in fats, compound the already high digestive process-generated availability of glucose by eliciting insulin resistance. The large excess of glucose thus generated is largely used to promote energy-consuming processes and may result, in a significant part, converted to lipid for storage; or, in the case of coexistent high dietary fat, used for immediate disposal to prevent hyperglycemia. But even this process has limits and excess glucose damages the liver-adipose tissue energy-maintenance axis, which extends to the whole body because of the implication of defense mechanisms that inadequately try to prevent these damages. The consequences are inflammation and the development of the MS.

## List of abbreviations

WAT: white adipose tissue; BAT: brown adipose tissue; MS: metabolic syndrome

## Competing interests

The author declares that he has no competing interests.
